# Adding Enzalutamide to First-Line Treatment for Metastatic Hormone-Sensitive Prostate Cancer: A Cost-Effectiveness Analysis

**DOI:** 10.3389/fpubh.2021.608375

**Published:** 2021-02-09

**Authors:** Peng-Fei Zhang, Dan Xie, Qiu Li

**Affiliations:** ^1^Department of Medical Oncology, Cancer Center, West China Hospital, Sichuan University, Chengdu, China; ^2^West China Biomedical Big Data Center, West China Hospital/West China School of Medicine, Sichuan University, Chengdu, China; ^3^Prenatal Diagnosis Center, Department of Obstetrics and Gynecology, West China Second University Hospital, Sichuan University, Chengdu, China; ^4^Key Laboratory of Birth Defects and Related Diseases of Women and Children (Sichuan University), Ministry of Education, Chengdu, China

**Keywords:** prostate cancer, enzalutamide, cost-effectiveness, androgen deprivation therapy, first-line

## Abstract

**Background:** The aim of this study is to evaluate the pharmacoeconomic profile of adding enzalutamide to first-line treatment for metastatic, hormone-sensitive prostate cancer (mHSPC) from the US and Chinese payers' perspectives.

**Materials and Methods:** A Markov model with three health states: progression-free survival (PFS), progressive disease (PD), and death, was constructed. All patients were assumed to enter the model in the PFS state and transit according to the transition structure. Efficacy data were derived from the ENZAMET trial and Weibull distribution curves were modeled to fit the survival curves. Costs in the model included cost of drugs, best-supportive care (BSC), follow-up, tests, and adverse events (AEs)-related treatments. The primary endpoint of the study was incremental cost-effectiveness ratio (ICER). In addition, the impact of several key parameters on the results of the cost-effectiveness analysis was tested with one-way sensitivity analyses and probabilistic sensitivity analyses.

**Results:** Overall, ICERs were $430,933.95/QALY and $225,444.74/QALY of addition of enzalutamide to androgen deprivation therapy (ADT) vs. ADT from the US and Chinese payers' perspective, respectively. The most influential factors were the utility for the PFS state and the cost of enzalutamide. At the willingness-to-pay (WTP) thresholds of $100,000.00/QALY in the US and $28,988.40/QALY in China, the probability of adding enzalutamide to first-line treatment being a cost-effective option for mHSPC was 0%.

**Conclusions:** Based on the data from the ENZAMET trial and the current price of enzalutamide, adding enzalutamide to first-line treatment is not cost-effective for patients with mHSPC from the US and Chinse payers' perspectives.

## Introduction

Prostate cancer is the second most frequently diagnosed cancer and ranks the fifth in cancer-related death in men worldwide. It was estimated that almost 1.3 million new cases and 359,000 deaths occurred in 2018 (1). Most patients with prostate cancer are diagnosed with localized disease, however, 10–20% of the patients are expected to be diagnosed with locally advanced or metastatic disease, for whom the standard first-line treatment is androgen deprivation therapy (ADT) (2). Bilateral orchiectomy and luteinizing hormone-releasing hormone (LHRH) agonists/antagonists represent the two main methods for ADT (3, 4). Recent years, the efficacy and safety of adding docetaxel or abiraterone to ADT, have been investigated in the first-line treatment for advanced/metastatic prostate cancer. All these drugs were demonstrated to significantly prolong the survivals in patients with metastatic hormone-sensitive prostate cancer (mHSPC) (5–8).

Enzalutamide is an oral androgen receptor inhibitor, which is designed to solve acquired resistance to first-generation non-steroidal antiandrogens, including bicalutamide, nilutamide, and flutamide (9). In previous trials, enzalutamide has been demonstrated to prolong the survivals in metastatic castration-resistant prostate cancer (mCRPC) (10, 11). Encouraged by the significant benefit of enzalutamide in mCRPC, the effects of adding enzalutamide to first-line treatment that included testosterone suppression with or without early docetaxel was investigated in the ENZAMET trial based on the hypothesis that adding enzalutamide to first-line therapy would delay the emergence of castration resistance and thereby improve survivals (12). As expected, enzalutamide was associated with significantly longer progression-free survival (PFS) and overall survival (OS) than standard care in men with mHSPC receiving testosterone suppression, with or without early docetaxel.

Although enzalutamide has shown significant benefit in the first-line treatment for patients with mHSPC, addition of enzalutamide may also increase health-care expenditures, which results in difficult treatment decisions for patients, doctors and policy makers. Moreover, health-care expenditures are growing rapidly and have become a major public health concern worldwide recently (13, 14). Thus, evaluation of a novel treatment options from both efficacy and pharmacoeconomic profile is of great significance. The aim of this study is to evaluate the cost-effectiveness of adding enzalutamide to first-line treatment in men with mHSPC from the US and Chinese payers' perspectives.

## Materials and Methods

### Model Structure

**Figure 1:**
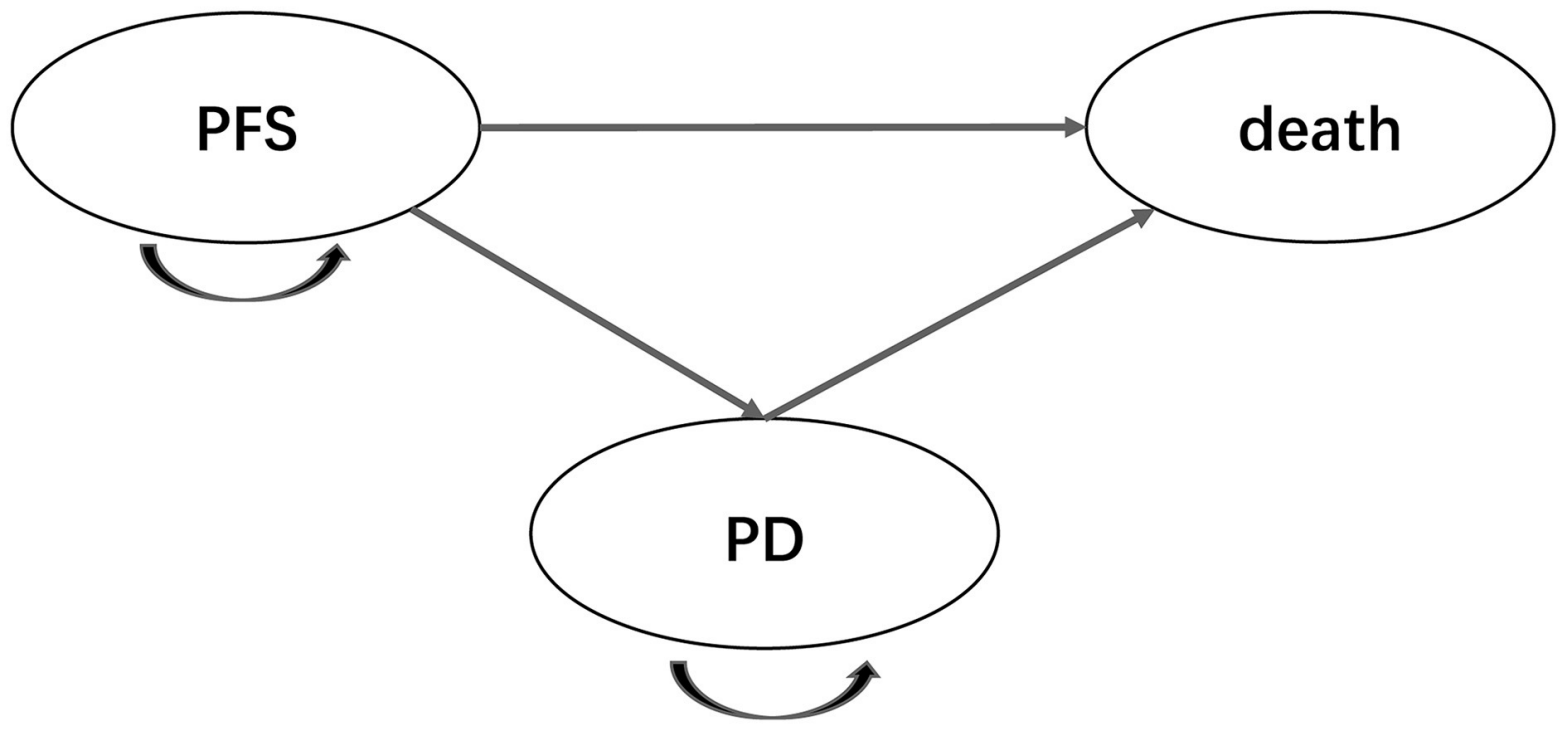
Markov model diagram for patients with metastatic, hormone-sensitive prostate cancer. PFS, progression-free survival; PD, progressive disease.

To investigate the cost-effectiveness of adding enzalutamide to first-line treatment for patients with mHSPC compared to standard care, a Markov decision model, which included three health states [PFS, progressive disease (PD) and death], was constructed. In the model, all patients were assumed to enter in the PFS state and then transit from one state to another state according to the transition structure presented in Figure 1. Time horizon of the model was defined as 20 years, after which almost all patients were expected to be dead. Markov cycle length was set at 1 month, which was consistent with the length of treatment periods. Health utilities for the PFS state and the PD state were derived from previous literature (Table 1) (15, 16). Health utility values in the model were assumed to be invariable despite the impact of AEs. Incremental cost-effectiveness ratio (ICER) was regarded as the primary endpoint of the study. Willingness-to-pay (WTP) threshold in the analysis was set at $28,988.40/quality-adjusted life year (QALY) (3 × per capita GDP of China, 2018) for China and $100,000.00/QALY for US, respectively. Cost and effectiveness were discounted at an annual rate of 3% for China and 3.5% for US, in line with the guidelines. This study was approved by the Ethics Committee of West China Hospital, Sichuan University. The model was developed and performed using the Microsoft Excel (Microsoft Corporation, Redmond, WA, USA) and TreeAge software (TreeAge, Williamstown, MA, USA, 2011).

**Table 1:** Key clinical data in the model.

**Parameters**	**Enzalutamide**	**Standard care**	**References**
**Survival data**			
OS at 3 years	80%	72%	(12)
PSA PFS at 3 years	67%	37%	(12)
Clinical PFS at 3 years	68%	41%	(12)
HR for death	0.67	-	(12)
HR for PSA PFS	0.39	-	(12)
HR for clinical PFS	0.40	-	(12)
**Grade 3–4 AEs (n, %)**			
Febrile neutropenia	37 (7)	32 (6)	(12)
Hypertension	43 (8)	25 (4)	(12)
Neutrophil count decreased	31 (6)	16 (3)	(12)
Fatigue	31 (6)	4 (1)	(12)
**Utility (China)**			
PFS	0.76 (0.608–0.912)	0.76 (0.608–0.912)	(15)
PD	0.68 (0.544–0.816)	0.68 (0.544–0.816)	(15)
**Utility (U.S.)**			
PFS	0.83 (0.664–0.996)	0.83 (0.664–0.996)	(16)
PD	0.725 (0.58–0.87)	0.725 (0.58–0.87)	(16)

*OS, overall survival; PSA, prostate specific antigen; PFS, progression-free survival; HR, hazard ratio; AEs, adverse events*.

### Patients and Treatments

A cohort population, which reflected the participant population of the ENZAMET trial, were modeled. Enzalutamide was administered at a dose of 160 mg daily. Participants in the standard care group received a conventional non-steroidal antiandrogen (i.e., bicalutamide 50 mg daily, nilutamide 150 mg daily, or flutamide 250 mg three times a day). All participants in the ENZAMET trial received standard background therapy, including LHRHA (goserelin, leuprorelin, triptorelin, and degarelix) or surgical castration. For patients who received docetaxel, docetaxel should be administered at 75 mg/m^2^ every 21 days for a maximum of 6 cycles.

### Efficacy and Cost Inputs

**Figure 2:**
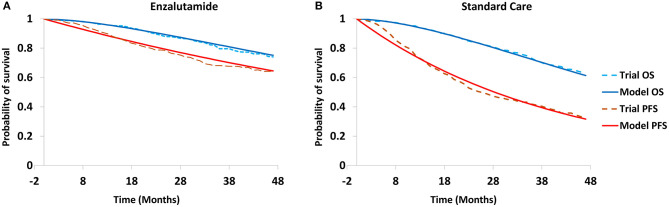
Modeled survival curves for enzalutamide group **(A)** and standard care group **(B)**. PFS, progression-free survival; OS, overall survival.

Efficacy data in the model were derived from the ENZAMET trial, and these data were used to estimate the transition probabilities between health stages (Table 1). Survival data were collected from the survival curves using the Web Plot Digitizer software (https://apps.automeris.io/wpd/). The estimated transition probabilities were modeled to fit the survival curves (Figure 2). To simplify the model, only AEs with a frequency ≥5% were included. The cost in the model were categorized into cost of drug, best-supportive care (BSC), follow-up (reflect the frequency of drug administration), tests and adverse events (AEs)-related treatments. The unit prices of drugs and tests in China were retrieved from the national drug prices or West China Hospital, Sichuan University, China. For analysis from the US payers' perspective, the unit prices of drugs were obtained from RED BOOK Online®, and unit cost of tests, drug administration, follow-up, tests, AEs-related treatments and BSC were retrieved from the CMS clinical laboratory fee schedule files and previously published literatures (Table 2) (17–23). To calculate the dose of docetaxel, participants with a body surface area of 2.1 or 1.72 m^2^ were assumed to reflect the patients in US and China (16, 24). All cost was converted into US dollars.

**Table 2:** Cost parameters input in the model.

**Parameters**	**Value ($)**	**Range**	**Resource**
Enzalutamide (40 mg)	48.61 (China)	38.888–58.332	Local estimate
	115.486 (US)	92.389–138.583	RED BOOK
Bicalutamide (50 mg)	6.04 (China)	4.832–7.248	Local estimate
	19.826 (US)	15.861–23.791	RED BOOK
Flutamdie (125 mg)	1.28 (China)	1.024–1.536	Local estimate
	2.939 (US)	2.351–3.527	RED BOOK
Nilutamide (150 mg)	-	**-**	**-**
	261.909 (US)	209.527–314.291	RED BOOK
Docetaxel (20 mg)	169.1 (China)	135.280–202.920	Local estimate
	187.576 (US)	150.061–225.091	RED BOOK
Goserelin (3.6 mg)	321.43 (China)	257.144–385.716	Local estimate
	740.52 (US)	592.416–888.624	RED BOOK
Leuprolide (3.75 mg)	195.83 (China)	156.664–234.996	Local estimate
	1560.44 (US)	1248.352–1872.528	RED BOOK
Triptorelin (3.75 mg)	311.68 (China)	249.344–374.016	Local estimate
	975.89 (US)	780.712–1171.068	RED BOOK
Histrelin (50 mg)	-	-	-
	23908.415 (US)	19126.732–28690.098	RED BOOK
Degarelix (80 mg)	435.22 (China)	348.176–522.264	Local estimate
	586.14 (US)	468.912–703.368	RED BOOK
Orchidectomy	83.11 (China)	66.488–99.732	Local estimate
	13194 (US)	10555.2–15832.8	RED BOOK
Laboratory tests	30.68 (China)	24.544–36.816	Local estimate
	76 (US)	60.8–91.2	(17)
PSA test	6.95 (China)	5.56–8.34	Local estimate
	25 (US)	20–30	(18)
CT	211.56 (China)	169.248–253.872	Local estimate
	828 (US)	662.4–993.6	(17)
Bone scan	141.34 (China)	113.072–169.608	Local estimate
	253.46 (US)	202.768–304.152	(18)
Febrile neutropenia	953 (China)	762.4–1143.6	(19)
	18507 (US)	14805.6–22208.4	(16)
Hypertension	12.15 (China)	9.72–14.58	(20)
	201.9 (US)	161.52–242.28	(21)
Neutrophil count decreased	466 (China)	372.8–559.2	(19)
	5937 (US)	4749.6–7124.4	(16)
Fatigue	108.73 (China)	86.984–130.476	(20)
	139 (US)	111.2–166.8	(21)
Cost of supportivecare per cycle	117.1 (China)	93.68–140.52	(22)
	1213 (US)	970.4–1455.6	(21)
Routine follow-up of patients per unit	51.5 (China)	41.2–61.8	(23)
	422 (US)	337.6–506.4	(21)

*CT, computed tomography*.

### Sensitivity Analysis

The robustness of the analysis was tested with a series of one-way sensitivity analyses on several parameters, such as duration of PFS, duration of OS, cost of enzalutamide. In the one-way sensitivity analyses, parameters ranged between ±20%, and tornado diagrams were used to display the results of the one-way sensitivity analyses. Moreover, probabilistic sensitivity analyses were also performed based on the Monte Carlo simulations with 1,000 iterations.

## Results

### Base Case Analysis

The results of the base case analysis were shown in Table 3. Over a lifetime horizon of 20 years, adding enzalutamide to first-line treatment gained an effectiveness of 6.21 QALYs at a cost of $1,396,827.63 from the US payers' perspective, while from the Chinese payers' perspective, the effectiveness and cost were 5.70 QALYs and $516,510.76. On the other hand, the cost and effectiveness in the standard care group were $483,247.66, 4.09 QALYs and $83,656.86, 3.78 QALYs from the US payers' perspective and the Chinese payers' perspective, respectively. Overall, ICERs were $430,933.95/QALY and $225,444.74/QALY of adding enzalutamide to first-line treatment vs. standard care from the US payers' perspective and the Chinese payers' perspective, indicating adding enzalutamide to first-line treatment was not cost-effective vs. standard care at the WTP threshold of $28,988.40/QALY in China and $100,000.00/QALY in the US.

**Table 3:** Base case results of the model.

**Model outcomes**	**Enzalutamide**	**Standard care**
**US**		
Total costs ($)	1,396,827.63	483,247.66
Incremental costs	913,579.97	-
Total effectiveness (QALYs)	6.21	4.09
Incremental effectiveness (QALYs)	2.12	-
ICER ($/QALY)	430,933.95	-
**China**		
Total costs ($)	516,510.76	83,656.86
Incremental costs	432853.90	-
Total effectiveness (QALYs)	5.70	3.78
Incremental effectiveness (QALYs)	1.92	-
ICER ($/QALY)	225,444.74	-

*QALY, quality-adjusted life year; IECR, incremental cost-effectiveness ratio*.

### Sensitivity Analyses

**Figure 3:**
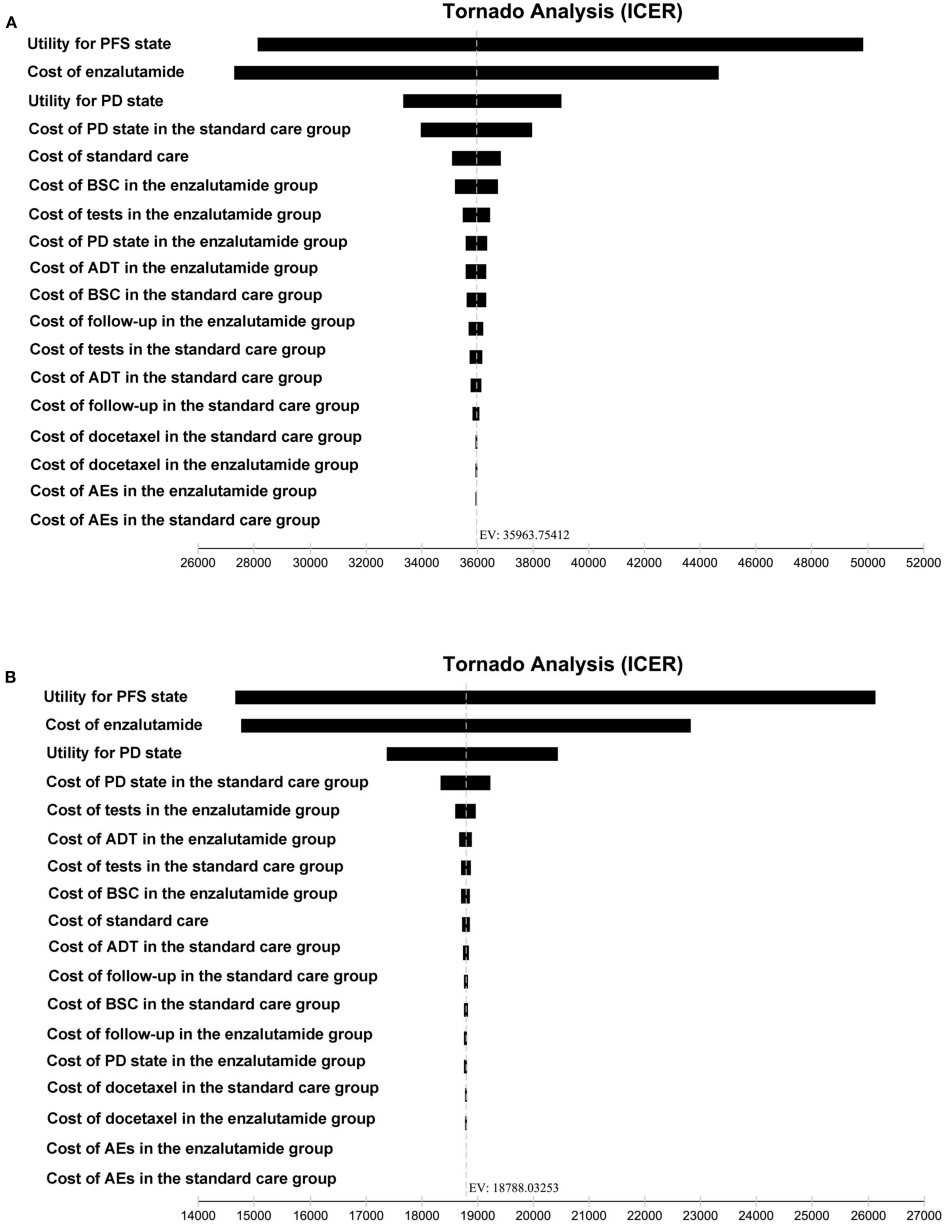
Tornado diagram for one-way sensitivity analyses. **(A)** The US payers' perspective. **(B)** The Chinese payers' perspective. PFS, progression-free survival; PD, progressed disease; BSC, best-supportive care; ADT, androgen-deprivation therapy; AEs, adverse events; ICER, incremental cost-effectiveness ratio.

To investigate the impact of key variables on the robustness of our results, one-way sensitivity analyses were performed, and the results of the one-way sensitivity analyses were presented in a tornado diagram. As shown in Figure 3, the utility for the PFS state and the cost of enzalutamide were the most influential factors both from the US payers' perspective and the Chinese payers' perspective.

We also performed a probabilistic sensitivity analysis to assess the uncertainty of the resultant ICER based on the Monte Carlo simulations with 1,000 iterations. The probabilities of adding enzalutamide to first-line treatment or standard care as the cost-effective option were 0 and 100% both from the Chinese and US payers' perspectives at the WTP threshold set in the model.

## Discussion

Prostate cancer represents one of the most commonly diagnosed cancer in men around the world. For locally advanced or metastatic diseases, ADT was previously considered as the standard first-line treatment. However, more and more novel drugs, such as docetaxel or abiraterone, were demonstrated to significantly improved the survivals in mHSPC. Recently, the results of the ENZAMET trial, which investigated the effect of adding enzalutamide to first-line treatment, was released. Consistent with the significant benefit achieved in mCRPC, adding enzalutamide to first-line treatment also significantly prolonged the survivals in patients with mHSPC. However, the price of enzalutamide was much higher than first-generation non-steroidal antiandrogens (bicalutamide, nilutamide, and flutamide). Thus, in this study, we evaluated the pharmacoeconomic profile of adding enzalutamide to first-line treatment in men with mHSPC from the US and Chinese payers' perspectives. Based on the analysis, the ICERs were $430,933.95/QALY and $225,444.74/QALY of adding enzalutamide to first-line treatment vs. standard care from the US payers' perspective and Chinese payers' perspective, respectively. From the pharmacoeconomic profile, adding enzalutamide to first-line treatment was not a cost-effective treatment option based on the data from the ENZAMET trial and the current price of enzalutamide.

Recently, a series of novel treatment options have been approved for the treatment of mHSPC. Despite the significant benefits achieved by these treatment options, novel treatment regimens may also cause heavy burden of health-care expenditures. To provide more evidences for selecting the optimal treatment regimen, more and more cost-effectiveness analyses were conducted to evaluate novel treatment regimens from the pharmacoeconomic profile. However, the conclusion of cost-effectiveness analysis in different regions were inconsistent. For example, two Chinese studies demonstrated that docetaxel combined with ADT is not a cost-effective treatment compared with ADT alone for mHSPC in the Chinese setting (25, 26). However, in the similar studies based on the US and Brazil, the addition of docetaxel with ADT in high-volume metastatic HSPC appears to be an economically attractive treatment approach (27, 28). Thus, in this study, we evaluated the cost-effectiveness of enzalutamide plus first-line treatment in men with mHSPC from both the US and Chinese payers' perspectives, which could extend the application of the results of the analysis. Our analysis, based on current costs and the results of ENZAMET trial after a median follow-up of 3 years, indicated that adding enzalutamide to first-line treatment is unlikely to be a cost-effective regimen from both the US and Chinese payers' perspectives.

Although adding enzalutamide to first-line treatment is not a cost-effective option treatment both in the US and China, we wonder whether the factors affecting the model are the same from the two perspectives. Thus, we performed a series of one-way sensitivity analyses to investigate the impact of key variables on the robustness of our results. The utility for the PFS state and the cost of enzalutamide were the most influential factors from the US payers' perspective. Meanwhile, the model was also sensitive to the utility for the PD state and the cost of PD state in the standard care group. The most influential factors from the Chinese payers' perspective were the same with that from the US payers' perspective. Moreover, the utility for the PD state and the cost of PD state in the standard care group were also another two factors mostly influencing the model.

This is the first study to assess the cost-effectiveness of enzalutamide in the first-line treatment of mHSPC, however, the pharmacoeconomic profile of enzalutamide has been evaluated in mCRPC. In a study, Pollard et al. performed a cost-effectiveness analysis to compare the contemporary treatment options for mCRPC, which shows that all currently available treatment options, including enzalutamide, exceed the commonly used WTP threshold of $100,000 per life year saved. In another study, Barqawi et al. evaluated the cost-effectiveness of abiraterone plus prednisone, cabazitaxel plus prednisone and enzalutamide for visceral mCRPC after docetaxel therapy resistance. Based on the cost-effectiveness analysis, enzalutamide was cost-effective in 92% of the time with a WTP threshold of $100,000/QALY for patients with visceral mCRPC after docetaxel therapy resistance (29, 30). The differences between the two study could be interpreted as the comparators were different. In Pollard's study, the standard care is docetaxel, which is relatively cheap. However, in Barqawi's study, abiraterone and cabazitaxel were also with high prices, which made enzalutamide as a cost-effective option.

The limitations of the study should also be addressed. First, the analysis was conducted based on the ENZAMET trial, and we merely investigated the cost-effectiveness of adding enzalutamide to first-line treatment vs. standard care for patients with mHSPC. We did not include other novel treatment options, such as abiraterone, as there are no head-to-head trials to compare the effect of these novel regimens. Second, the analysis included AEs with relatively frequent (>5%), expensive to treat or substantively affected quality of life. The cost of grade 1–2 AEs and AEs with low frequency were not included in the study. Fortunately, the results of the one-way sensitivity analyses demonstrated the economic results were not sensitive to AEs-related parameters. Third, several key parameters, such as the utility scores, cost of AE-related treatments, and cost of supportive care in the analysis, were derived from previously published literature, which may also undermine the correctness of our results. We believe that cost-effectiveness analysis based on real-world population may be more correct and representative.

## Conclusions

In this study, we evaluated the pharmacoeconomic profile of adding enzalutamide to first-line treatment in men with mHSPC from the US and Chinese payers' perspectives. Based on the WTP threshold of $28,988.40/QALY for China and $100,000.00/QALY for US, enzalutamide, at its current price and based on 3-year follow-up from ENZAMET trial, is not cost-effective compared with standard care from the US and Chinse societal perspectives.

## Data Availability Statement

The data generated during this study are available from the corresponding author on reasonable request.

## Author Contributions

P-FZ and QL were responsible for the study conception, methodology, data analysis, draft writing, and editing. DX was responsible for the design, data collection and analysis, draft writing and editing. All authors contributed to the article and approved the submitted version.

## Conflict of Interest

The authors declare that the research was conducted in the absence of any commercial or financial relationships that could be construed as a potential conflict of interest.
